# Characterization and use of the ECV304 autoantigenic citrullinome to understand anti-citrullinated protein/peptide autoantibodies in rheumatoid arthritis

**DOI:** 10.1186/s13075-021-02698-2

**Published:** 2022-01-13

**Authors:** Natalia Regine de França, Henri André Ménard, Maximilien Lora, Zhijie Zhou, Joyce Rauch, Carol Hitchon, Luís Eduardo Coelho Andrade, Inés Colmegna

**Affiliations:** 1 Division of Rheumatology, Department of Medicine, McGill University, The Research Institute of the McGill University Health Centre, 1001 Décarie Boulevard, Montréal, QC H4A 3J1 Canada; 2 Division of Rheumatology, Paulista School of Medicine, Federal University of Sao Paulo, Sao Paulo, Brazil; 3 Section of Rheumatology, Department of Medicine, University of Manitoba, Winnipeg, MB Canada

**Keywords:** ECV304, Autocitrullinome, Peptidylarginine deiminases, ACPAs, Rheumatoid arthritis

## Abstract

**Background:**

Anti-citrullinated protein antibodies (ACPAs) are highly specific for rheumatoid arthritis (RA). In vivo, ACPAs target peptidyl-citrulline epitopes (cit-) in a variety of proteins (cit-prot-ACPAs) and derived peptides (cit-pept-ACPAs) generated via the peptidylarginine deiminase (PAD) isoenzymes. We aimed to identify a cell line with self-citrullination capacity, to describe its autoantigenic citrullinome, and to test it as a source of autocitrullinated proteins and peptides.

**Methods:**

Human cell lines were screened for cit-proteins by Western blot. PAD isoenzymes were identified by RT-PCR. Autocitrullination of ECV304 was optimized, and the ECV304 autocitrullinomes immunoprecipitated by sera from three RA patients were characterized by mass spectrometry. Cit-pept-ACPAs were detected using anti-CCP2 ELISA and cit-prot-ACPAs, by an auto-cit-prot-ECV304 ELISA. Sera from 177 RA patients, 59 non-RA rheumatic disease patients and 25 non-disease controls were tested.

**Results:**

Of the seven cell lines studied, only ECV304 simultaneously overexpressed PAD2 and PAD3 and its extracts reproducibly autocitrullinated self and non-self-proteins. Proteomic analysis of the cit-ECV304 products immunoprecipitated by RA sera, identified novel cit-targets: calreticulin, profilin 1, vinculin, new 14–3-3 protein family members, chaperones, and mitochondrial enzymes. The auto-cit-prot-ECV304 ELISA had a sensitivity of 50% and a specificity of 95% for RA diagnosis.

**Conclusions:**

ECV304 cells overexpress two of the PAD isoenzymes capable of citrullinating self-proteins. These autocitrullinated cells constitute a basic and clinical research tool that enable the detection of cit-prot-ACPAs with high diagnostic specificity and allow the identification of the specific cit-proteins targeted by individual RA sera.

**Supplementary Information:**

The online version contains supplementary material available at 10.1186/s13075-021-02698-2.

## Background

Rheumatoid arthritis (RA) is a chronic polyarthritis where ACPAs define a major subset (50 to 75%) of patients, with an increased risk of severe disease course [1–3]. ACPAs represent a heterogeneous group of antibodies recognizing a variety of cit-epitopes either on proteins (cit-prot-ACPAs) or peptides (cit-pept-ACPAs) [4]. ACPAs in the ACR-EULAR 2010 RA classification criteria refer to the latter i.e., anti-cyclic-citrullinated-peptide 2 (CCP2) ELISA [5]. Studies of human ACPA specificities show that anti-CCP2 ACPAs are not cit-protein specific and target widely cross-reactive epitopes on cit-peptides/cit-proteins candidates [6]. According to a structural model defining their immune interactions [7], most cit-pept-ACPAs target so-called “promiscuous” cit-epitopes for which there is no conclusive evidence of a pathogenic role [8]. In contrast, cit-prot-epitopes designed as “private” are defined by the amino acids neighbouring citrulline residues on the targeted autoantigenic cit-protein. As pathogenic autoantibodies in RA should target “private” cit-prot-epitopes in the joints, those epitopes would be ideally detected using whole cit-proteins [8, 9].

Several cit-prot-autoantigens with potential pathophysiologic roles have been proposed [10, 11]. However, to date only few studies used whole cit-prot autoantigens in a systematic manner to detect cit-prot-ACPAs [12, 13]. Here, we characterize the cit-protein autocitrullinome of ECV304 and show that it is a useful substrate to screen for and, define specific ACPAs-proteins interactions.

## Methods

### Cell lines and lysates

A convenience sample of seven human cell lines from American Type Culture Collection (ATCC) were tested: ECV304 (ATCC® CRL-1998™ derivative of T24), T24 (ATCC® HTB-4™, urinary bladder epithelial carcinoma), BJ (ATCC® CRL-2522™, normal foreskin fibroblast), HCA-2 (colon epithelial adenocarcinoma), HEK293T (embryonic kidney epithelial cells), PC-3 (prostate epithelial adenocarcinoma), and HeLa (cervix epithelial adenocarcinoma). To prevent PAD activation, cells collected mechanically from monolayers were washed with TEN solution (40 mM Tris–Cl, pH 7.5, containing 1 mM EDTA and 0.15 M NaCl). Cell lysates were prepared by three freeze–thaw cycles. Post-centrifugation, the protein concentration in the cell lysates supernatant, hereafter ‘cell lysate’, was measured (Bradford protein assay, Bio-Rad, Hercules, CA, USA), aliquoted and stored at -80˚C.

### Optimization of in vitro citrullination

Cell lysates [0—50 µg] were incubated in citrullination buffer [5 mM dithiothreitol (DTT), 0.1 M Tris–HCl, 10 mM CaCl_2_] [14] at 37˚or 55˚C for defined time periods (up to 24 h) to generate optimal autocitrullinated lysates (CIT). Cell lysates were similarly incubated without calcium (non-citrullinated control lysates—CTR). Citrulline was quantified by a colorimetric assay [15].

### Western blots (WB) of citrullinated proteins

Denaturing 12% SDS-PAGE conditions were used. Gels were transferred to polyvinylidene fluoride (PVDF) membranes (Amersham Hybond-P 0.45 nm). To detect citrullinated proteins a commercial kit was used: Anti-Citrulline (modified) Detection Kit (UPSTATE Lake Placid NY USA). Citrulline residues on the blot were chemically modified by overnight incubation in the modification medium (0.0125% FeCl_3_, 2.3 M H_2_SO_4_, 1.5 M H_3_PO_4_, 0.25% diacetyl monoxime, 0.125% antipyrine, and 0.25% acetic acid) at 37 °C [16]. Before the membranes were probed with anti-chemically modified citrulline (0.125 ug/ml) for 3 h, the blot was rinsed several times with water and then soaked in blocking buffer (5% powdered milk in PBS) for 1 h. After three washes with PBS, peroxidase-conjugated goat anti-rabbit IgG (Jackson Immuno Research laboratories, Inc. West Grove, Pennsylvania) was added, and the membranes were developed using the ECL system. To detect antigenic proteins the membrane was blocked in 5% non-fat powder milk in PBS and incubated overnight (4˚C) with RA sera (1/200 dilution). After washing (PBS/Tween 0.1%), membranes were incubated [1 h, room temperature (RT)] with horseradish peroxidase-conjugated goat anti-human IgG (Jackson Immunoresearch Laboratories, Inc.) diluted 1/500 in blocking solution. β-actin (c4) antibody (Santa Cruz Biotechnology, Dallas, TX, USA) diluted 1/10000 was used as loading control. To confirm the presence of vimentin, anti-vimentin, monoclonal clone V9 (Sigma-Aldrich, St. Louis, MO, USA) antibodies diluted 1/500 were added (4˚C overnight incubation). Membranes were developed with the Amersham ECL Prime WB Detection Reagent (GE Healthcare). The Omega Lum C Image Capture Software was used for analysis (Aplegen, San Francisco, CA, USA).

### Identification and expression of PAD isoenzymes

Cell lysates were kept in Trizol (Thermo Fisher Scientific, Waltham, MA, USA) at -80˚C until RNA isolation (Direct-zol RNA MiniPrep, Zymo Research, Irvine, CA, USA). cDNA was synthetized from 1 µg of RNA with QuantiTech reverse transcription kit (QIAGEN, Hilden, Germany). For PCR, 2 µL cDNA template was added to 25 µL RedTaq® ReadyMix™ PCR Reaction Mix (Sigma Aldrich, Sigma Aldrich, St. Louis, MO, USA) containing 0.5 mM of forward and reverse primers. PCR was performed using thermocycler Mastercycler Pro Thermal Cyclers Pro S (Eppendorf, Hamburg, Germany): one minute at 94 °C; 35 cycles (each: 60 s, 94 °C; 2 min, 58 °C; 3 min, 72 °C); and 10 min at 72 °C. PCR products (50 µL) were analyzed by electrophoresis on a 1.5% agarose gel using the Mini Sub™ DNA Cell (Bio-Rad, Hercules, CA, USA). Primer pairs (size of the amplicon) (Gibco/BRL, Rockville, MD, USA) for each PAD isoenzymes and size of the amplicon are presented in Table S1.

### Immunoprecipitation (IP) and proteomic analysis

A protein G immunoprecipitation (IP) kit (Sigma-Aldrich, St. Louis, MO, USA) was used. Briefly, 200 µg of ECV304 cell lysates (without the addition of any exogenous proteins) either CIT (18 h at 55˚C) or CTR were incubated with 5 µL of three different ACPA positive RA sera for 1 h at 4˚C with protease inhibitors (Millipore Sigma, Ontario, Canada), IP buffer, and protein G beads. Immunoprecipitated proteins were submitted for proteomic analysis at the Research Institute of the McGill University Health Centre (RI-MUHC) Clinical Proteomics Platform [17]. A single stacking gel band was reduced with DTT, alkylated with iodoacetic acid, and digested with trypsin. Peptides were re-solubilized in 0.1% aqueous formic acid/2% acetonitrile and loaded onto a Thermo Acclaim Pepmap precolumn (Thermo; 75 µM ID X 2 cm C18 3 µM beads) followed by an Acclaim Pepmap Easyspray analytical column (Thermo, 75 µM X 15 cm with 2 µM C18 beads). Separation was performed using a Dionex Ultimate 3000 µHPLC at 220 µl/min with a gradient of 2–35% organic (0.1% formic acid in acetonitrile) over 3 h. Peptides were analyzed using a Thermo Orbitrap Fusion mass spectrometer (120,000 resolution; FWHM in MS1, 15,000 FWHM for MS/MS), with higher-energy collisional dissociation (HCD) to sequence all peptides with a charge of 2 + or greater. Raw data was converted into *.mgf format (Mascot generic format) and searched against human sequences (Swissprot 2017) using Mascot 2.3. The database search results were loaded onto Scaffold Q + Scaffold 4.7.2 (Proteome Sciences, Portland, OR, USA) for spectral counting, analysis (protein threshold 99%, minimal number of peptides 2, and peptide threshold 95%) and for deamination detection. Citrullinated peptides were automatically detected using a + 0.9840 atomic mass units (amu) modification to Arginine (< 5 ppm mass error in MS1). Deamidation of asparagine or glutamine (also + 0.9840 amu) were also included in the MS/MS searches to avoid misassignment of modified residues. Furthermore, trypsin cleavage was blocked when arginine was converted to citrulline. This combination of high mass accuracy in MS1 and MS/MS, high quality/accuracy MS/MS assignments of the + 0.9840 amu addition to the arginine residue, controlling for deamination of asparagine/glutamine to avoid forcing a mass error onto the wrong residues, and the trypsin miscleavage rule provided high confidence to the site-specific assignment of citrullination modifications in proteins [18]. Circos was used for data visualization of citrullinome [19].

### Cit-ECV304 ELISA

Using optimized citrullination conditions (55˚C for 18 h), ECV304 cell lysates alone were prepared. Briefly, 200 µg of ECV304 cell lysates either CTR or CIT (18 h at 55˚C) were prepared and 50 ug of the lysate was used to verify the quality of the citrullination by colorimetric assay. The remaining CTR and CIT lysates were used to prepare the ELISA plates (Maxisorp, NUNC, Thermo Fisher Scientific). The plates were coated overnight at 4˚C with 1 µg/100 µl/well of CTR (48 wells) or CIT (48 wells) ECV304 lysates in 0.1 M NaHCO_3_ pH 9.5. The plates were washed with PBS/Tween 0.05%, blocked with 1% BSA/PBS (1 h, RT) and incubated with sera (1/300 dilution) (3 h, RT, duplicates). The plates were washed and developed with alkaline phosphatase-conjugated goat anti-human IgG, diluted 1/1000 according to the manufacturer`s instructions (Sigma). The net binding to cit-proteins (i.e., CIT OD_405_ – CTR OD_405_) was reported.

### Patients and sera

Sera were obtained from 177 RA patients, 59 with non-RA rheumatic diseases and 25 healthy controls attending the MUHC or the University of Manitoba rheumatology clinics. Sera were stored in aliquots at -20˚C and thawed only once. RA patients fulfilled the ACR-EULAR 2010 classification criteria [5]. This study was approved by the MUHC Ethics Review Board (MP-37–2017-2773; 1992–810, 91–06-04; GEN-09–239) and the University of Manitoba Biomedical Research Ethics Board (HS 15,191 B2001:070). Sera from three different RA patients were used for IP. Anti-cit-pept-ACPAs were detected by anti-CCP2 ELISA (Euroimmun, Lübeck, Germany) and RF (rheumatoid factor) by nephelometry (Beckman Coulter, Pasadena, CA, USA).

### Statistical analysis

Statistical analysis was performed using IBM® SPSS Statistics (version 23.0; Armonk, NY, USA) and GraphPad Prism (version 8; San Diego, CA, USA). Normality of data distribution was tested by the Shapiro–Wilk test. Unless stated otherwise, error bars represent standard deviation (SD). Paired or unpaired 2-tailed Student’s tests were used for two groups comparisons and one-way ANOVA with Tukey’s post hoc test for multigroup comparisons. P-values less than 0.05 were considered significant. The optimal cut-off value of the ECV304 ELISA was defined by a ROC curve and the Youden index was used to support the cut-off selection for a positive test with high specificity for RA diagnosis [20]. Univariate and multiple logistic regression analyses were performed to test for associations between positive ECV304 ELISA and specific RA clinical features.

## Results

### ECV304: a cell line capable of self-citrullination

**Fig. 1:**
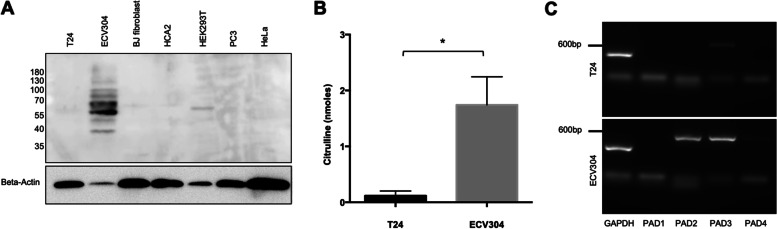
ECV304 produces high levels of citrullinated proteins. **A** WB of citrullinated proteins detected by RA serum in different cells lines. Total cell lysates were incubated under citrullination conditions followed by SDS-Page fractionation. Bands were revealed using an ACPA-positive RA serum and an anti-human IgG, HRP-conjugate. **B** Although T24 and ECV304 cell lines derive from the same individual, they differ in their citrulline content (*n* = 3, *p* < 0.05, unpaired T test). **C** Agarose gel electrophoresis depicting RT-PCR products for amplification of PAD enzyme isotype mRNA. ECV304 has higher mRNA levels of PAD2 and PAD3 isotypes

First, we investigated the presence of PAD activity capable of producing cit-proteins in the lysates of seven human cell lines and assessed cit-prot-epitopes by WB using a single RA serum with high anti-CCP2 titer. The CIT-ECV304 lysate was the only cell line in which the RA serum recognized multiple citrullinated proteins. A single faint band was observed in HEK293T lysate. No detectable citrullinated proteins were observed by Western blot of chemically modified citrulline in ECV304 cell lysate without calcium (Fig. S1). Surprisingly, no bands were found in T24, despite being from the same cell source as ECV304 (Fig. 1A). The authentication of our ECV304 and ATCC ECV304 CRL-1998 cell lines, and our T24 and ATCC T24 HTB-4 cell lines was done by short tandem repeat DNA profiling at ATCC (Table S2). There were differences in cit-content between ECV304 and T24 cells following the addition of calcium (ECV304: 1.74 ± 0.50 *versus* T24: 0.12 ± 0.08 nmoles, mean ± SD, *p* = 0.03) (Fig. 1B). Next, we assessed whether differences in the expression of PAD isotypes accounted for differences in citrullination. ECV304 expressed high levels of PAD2 and PAD3 mRNA but no detectable PAD1 or PAD4 mRNA. In contrast, T24 expressed only low levels of PAD3 mRNA (Fig. 1C). Therefore, PAD2 and PAD3 in ECV304 promote the citrullination of proteins recognized by antibodies in RA sera.

### ECV304 citrullinome

**Fig. 2:**
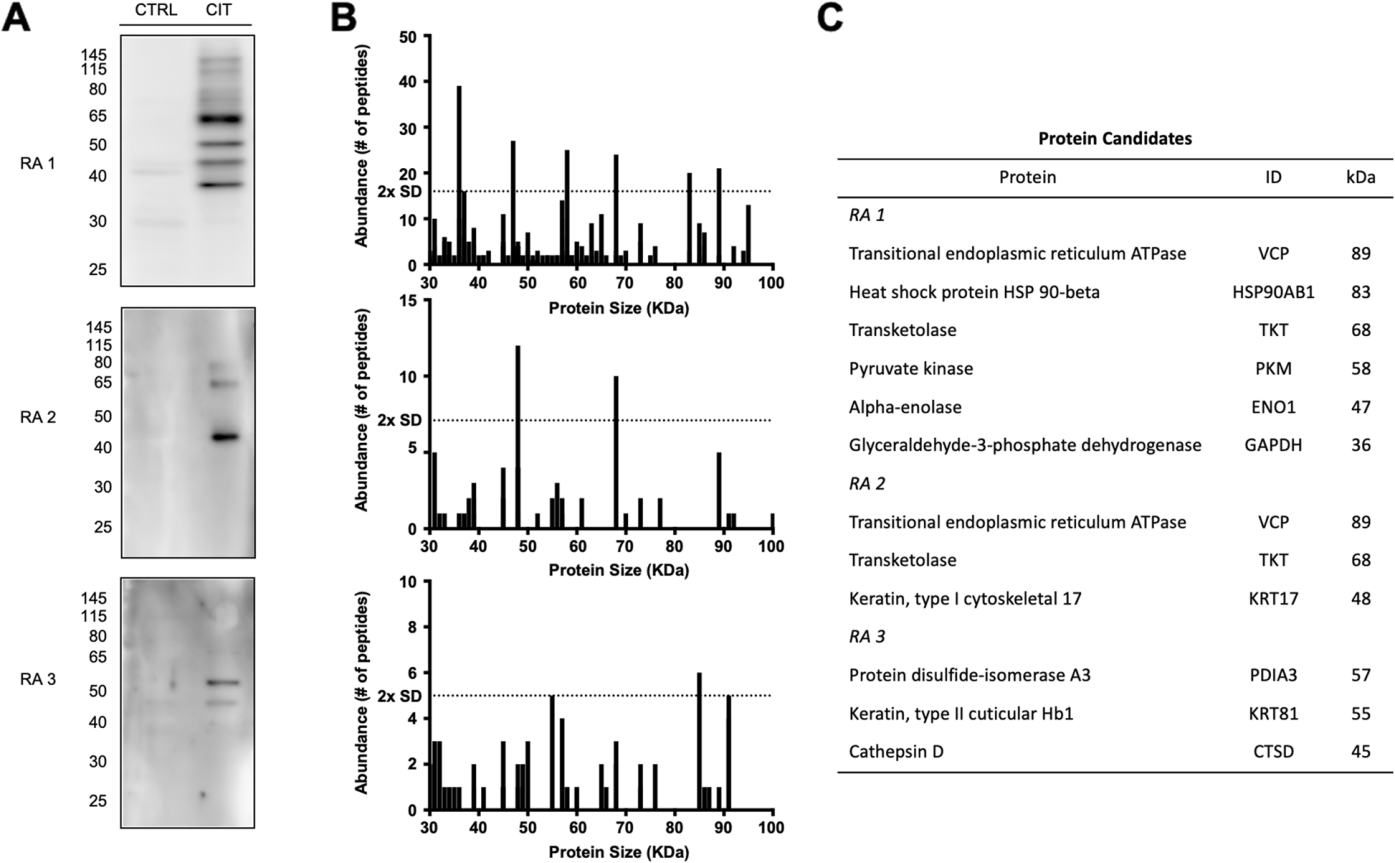
WB and proteomic analysis of immunoprecipitates using ECV304 citrullinated lysate and ACPA-positive RA sera. The size (kDa) of the proteins with the most abundant peptides (> 2SD) from the IP correlates with the molecular weight of the bands visualized on the WB (**A** and **B**). The protein candidates, their accession number and size are in **C**

**Fig. 3:**
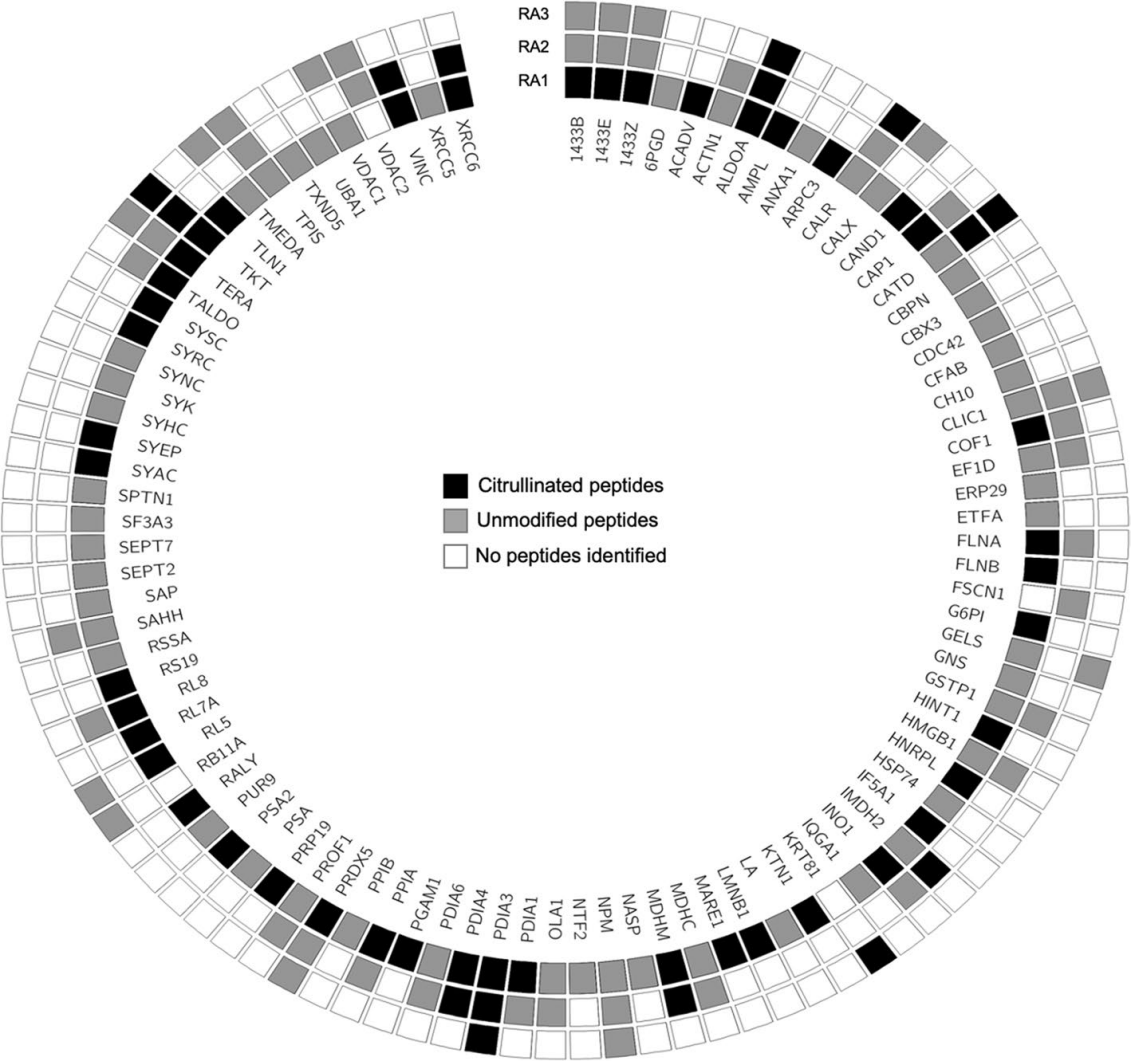
Autocitrullinome of ECV304. Proteins of citrullinated ECV304 cell lysates recognized by 3 RA sera (RA 1, RA 2, and RA 3). Citrullinated peptides are shown in black, unmodified peptides are shown in gray and proteins not detected by those specific RA sera are shown in white

Following the optimization of ECV304 autocitrullination conditions (lysate concentration 50 µg, temperature 55˚C, incubation time 18 h) we evaluated the composition of the ECV citrullinome. Three RA sera were selected based on their high anti-CCP titres (Table 1) and different reactivity in WB with ECV304 CTR or CIT lysate as substrates (Fig. 2A). Immune complexes were immunoprecipitated and characterized by mass spectrometry. A total of 353 proteins were identified in the immunoprecipitates. Fifteen were detected only in CTR, 241 in both CTR and CIT preparations and 97 proteins were unique to CIT preparation (Table S3). Among the 15 proteins uniquely immunoprecipitated in the CTR ECV304 cell lysate, only TRIM21 protein also known as Sjogren Syndrome Antigen A1 (52 kDa, Ribonucleoprotein Autoantigen SS-A/Ro) was detected by the 3 sera. Among the 241 proteins found in both immunoprecipitated CTR and CIT ECV304 cell lysate, 87 were immunoglobulins from the RA sera. Of interest, heat shock protein (Hsp) Hsp90α and Hsp90β although present in both preparations, had significantly more peptides captured in the CIT than in the CTR preparations. Citrullinated Hsp90α and citrullinated Hsp90β are proposed as autoantibody targets distinguishing RA patients with interstitial lung disease from those without lung disease [21]. Ninety-seven proteins constitute the antigenic self-citrullinome of ECV304 (Fig. 3). Of these proteins, 41 were detected by two of the three RA sera (Table S3—italic), and 24 were previously identified as autoantigens in rheumatic diseases. Thirteen proteins were recognized in all samples (Table S3—bold). When those proteins were interrogated specifically for Arg-Cit conversion, 76.9% (10 out of 13) were confirmed to have citrullinated peptides by mass spectrometry. Of interest, in the CIT fraction, the proteins with the most abundant peptides identified had molecular masses similar to the bands in the respective WB. Those include known and novel RA cit-autoantigens (Fig. 2 B, C).

**Table 1:** Clinical features of RA patients’ sera studied by immunoprecipitation

	RA1	RA2	RA3
Age (years) / Sex	64 / F	67 / F	50 / F
Disease duration (years)	13	16	29
Extra-articular manifestations	Nodules^a^, vasculitis	ILD^b^	Uveitis
RF (negative < 14 IU/mL)	349	1440	< 20
CCP (negative < 5 RU/mL)	> 200	176	> 200
ECV304 (negative < 0.044 delta OD)	0.547	0.294	0.065

^a^Subcutaneous nodules

^b^ *ILD* Interstitial lung disease

### RA autoantibodies are detected in the multi-cit-prot-ECV304 ELISA

**Fig. 4:**
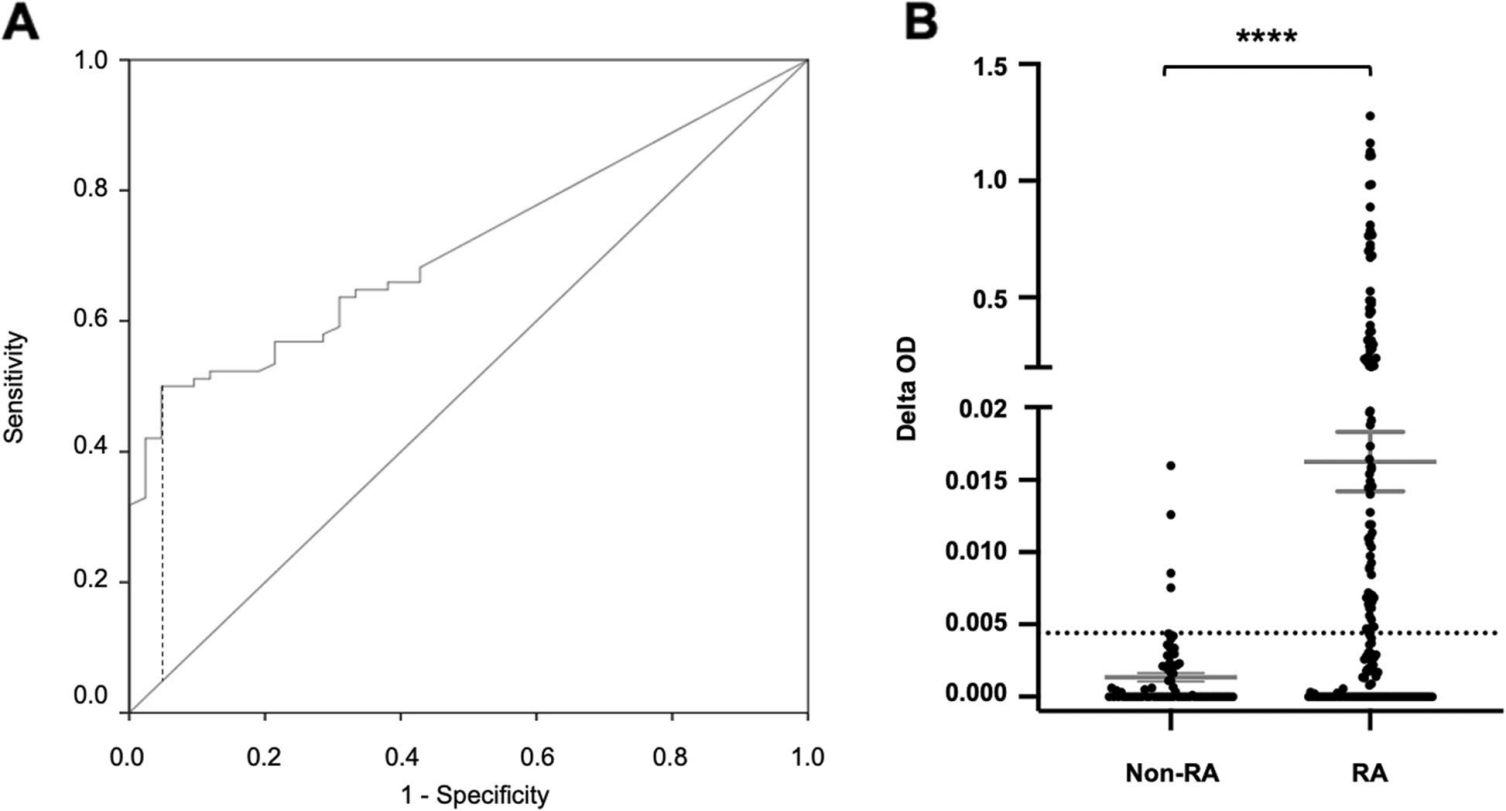
ECV304 ELISA for detection of autoantibodies in RA. **A** ROC curve, AUC of 0.716 (*p* < 0.001) and Youden index cut-off of 0.044, provided a sensitivity of 50% and specificity of 95.2%. **B** ECV304 ELISA scatterplot of non-RA (*n* = 84) and RA (*n* = 177) samples. The number (%) and titers (OD units as mean ± SE) of EVC304 positive sera were higher in RA with (48.6% positive and 0.163 ± 0.021 OD versus non-RA with 7.1% positive and 0.018 ± 0.004 OD (*p* < 0.0001 unpaired T test)

Sera from 177 RA patients [74.3% females (F), mean age ± SD: 56.7 ± 14 years], 59 patients with non-RA autoimmune rheumatic diseases: ANCA-positive vasculitis (*n* = 11, 36.4% F, 56.2 ± 18 years), systemic lupus erythematosus (*n* = 9, 77.8% F, 54.2 ± 14.4 years), Sjögren syndrome (*n* = 6, 83.1% F, 67.3 ± 7.2 years), psoriatic arthritis (*n* = 9, 66.7% F, 48.4 ± 15.7 years), enteropathic arthritis (*n* = 3, 33.3% F, 47 ± 20 years), osteoarthritis (*n* = 19, 73.7% F, 58.9 ± 15.1 years), undifferentiated arthritis (*n* = 1, F, age 64), dermatomyositis (*n* = 1, F, age 25) and 25 individuals without rheumatic or other chronic diseases (i.e. healthy controls) (68% F, 45 ± 12.2 years) were tested with ECV304 ELISA. The results were merged and randomly allocated to two equal groups. In group A (derivation), 88 RA and 42 controls (non-RA rheumatic diseases + healthy controls) were used to calculate the ELISA cut-off. Group B (validation) was similar in size with 89 RA and 42 controls (Table 2). A cut-off value for test positivity of 0.044 OD units (citrullinated OD_405_ – control OD_405_) in group A, provided 95% specificity, 50% sensitivity, 95.7% positive predictive value, 47.6% negative predictive value, 64.6% accuracy and a ROC area under the curve of 0.72 (SE 0.04, *p* < 0.001) (Fig. 4A) for RA. In group B, this cut-off provided a specificity of 90.5% (95% CI: 77.4—97.3%) and a sensitivity of 47.2% (95% CI: 36.5—58.1%). Overall, 48.6% of RA and 7.1% of non-RA patients had positive ECV304 ELISA tests (Fig. 4B).

**Table 2:** Demographics and clinical findings of RA patients

	Derivation (group A)		Validation (group B)	
	*n*	RA	*n*	RA
Age (years, mean ± SD)	87	56.1 ± 13.8	88	57.3 ± 14.2
Female (%)	87	64 (73.6)	88	66 (75)
Serology n (%)				
CCP positive	74	52 (70.3)	78	57 (73.1)
RF positive	82	48 (58.5)	85	53 (62.4)
CRP^b^ (mg/L)	63	10.91 ± 22.73	74	7.24 ± 11.86
Tobacco n (%)	85	22 (25.9)	82	19 (23.5)
Disease duration (years)	88	11.6 ± 5.8	89	10.3 ± 6.1
Active disease^a^ n (%)	81	42 (51.9)	82	37 (45.1)
RAPID-3 scores^c^	71	27 (38)	67	24 (35.8)
Erosions	82	27 (32.9)	84	25 (29.8)
Subcutaneous nodules	84	11 (13.1)	88	9 (10.2)
ILD^d^	87	4 (4.6)	89	3 (3.4)
Current treatment n (%)				
Biologics	86	36 (41.9)	85	33 (38.8)
DMARD^e^	85	78 (91.8)	86	78 (90.7)
Prednisone > 5 mg	85	6 (7.1)	85	7 (8.2)
Biologics ever	84	42 (50)	85	41 (48.2)

^a^Active disease: defined as presence of at least one swollen joint

^b^ *CRP* C-reactive protein, ^c^ *RAPID* routine assessment of patient index data, ^d^ *ILD* interstitial lung disease, ^e^ *DMARD* disease-modifying antirheumatic drug

In univariate logistic regression, CIT-ECV304 ELISA positivity was associated with CCP + (OR 4.03, 95% CI 1.92 – 8.46, *p* < 0.001), RF + (OR 2.94, 95% CI 1.53- 5.64, *p* < 0.001), presence of erosions (OR 2.2, 95% CI: 1.07 – 4.49, *p* < 0.03), and older age [each year increased the probability to be ECV304 + (OD 1.02 *p* = 0.036)]. There was a trend that the presence of subcutaneous nodules (OR 2.83, 95% CI: 0.90—8.87, *p* < 0.07) was associated with ECV304 positivity. In multiple logistic regression models, only CCP + (OR 3.38, 95% CI: 1.49 – 7.67, *p* < 0.004) and RF + (OR 2.95, 95% CI: 1.34 – 6.47, *p* < 0.007) were associated with ECV304 positivity.

## Discussion

ECV304 is a human cell line genetically identical to T24/83, a bladder carcinoma cell line [22]. Although initially reported as spontaneously transformed from HUVEC, ECV304 display different phenotypic characteristics than vascular endothelium (e.g. expression of calcitonin receptor) [22], and have epithelial markers such as desmoglein and cytokeratin similar than T24 [23]. On the other hand, ECV304 have endothelial markers including von Willebrand factor, receptors for low-density lipoprotein, and vimentin that are not in T24 cells [23]. ECV304 overexpress PAD2 and PAD3 and are capable of self-citrullination post-calcium activation in vitro. PAD3 is found in several cell types but mostly in epithelial cells, as is PAD2 which is also found in neuronal and myeloid cell types [24]. In contrast, T24 expresses PAD3 (although lower levels compared to ECV304) but not PAD2. The difference in PAD enzyme expression between ECV304 and T24 results in differential citrullination in the cells extracts (Fig. 1). It is precisely the ability of ECV304 to produce abundant citrullinated proteins that makes it a useful tool in the study of citrullinated antigens. Another example of a cell line not implicated in disease pathogenesis that is used as a diagnostic platform is the HEp-2 human epithelial cell tumor line [25].

The ECV304 autocitrullinome is recognized by ACPAs from RA patients and contains known and novel cit-targets. Previously described cit-prot-targets also identified by our analysis are calreticulin; fructose-bisphosphate aldolase A (an enzyme binding to intermediate filaments); mitochondrial enzymes (triosephosphate isomerase and α-enolase 1); and chaperones (14–3-3 protein family members, heat shock protein 90β and protein disulfide isomerases). The 14–3-3 protein family has seven isoforms (β, ε, γ, η, σ, τ, ζ), of which both native and cit-14–3-3η (not present in ECV304) are recognized RA autoantigens associated with accelerated joint damage [26]. We detected ACPAs targeting cit-epitopes on three other 14–3-3 family members: 14–3-3β/α, 14–3-3ε and 14–3-3ζ/δ. Native ε and ζ proteins were previously identified as autoantigens in sera from patients with large vessel vasculitis [27], but neither their native nor their cit-forms were known as RA ACPA targets. The 14–3-3 proteins are most important in various kinase signaling and regulatory pathways. Calreticulin is an autoantigen in RA [28] and other connective tissue diseases [29, 30]. Cit-calreticulin is abundant in RA synovial tissue and fibroblast-like synoviocytes where it is targeted by ACPAs. A cit-pept-calreticulin interacts with the HLA shared epitope with a 10,000-fold higher affinity than its corresponding native calreticulin peptide [31]. Enzymes linked to cellular metabolic functions are also cit-targets. In addition, loss of function of several cit-serpin targets have recently been described [10]. Autoantibodies (possibly unrecognized ACPAs) targeting so-called “denatured” fructose-bisphosphate aldolase A have been described in sera from erosive RA patients [32]. Similarly, anti-triosephosphate isomerase autoantibodies have been described in < 6% of RA samples [33] and anti-α-enolase 1 autoantibodies were found in 40% of RA patients [34]. Finally, anti-cit-heat shock protein 90β autoantibodies were proposed as biomarkers for RA interstitial lung disease [21]. All have been captured in the ECV304 autocitrullinome. This illustrates the multiplicity and complexity of the hidden part of citrullinated autoimmune systems in RA.

Four cit-autoantigen candidates need to be discussed in relation to our findings in the ECV304 autocitrullinome: cit-fibrin, cit-collagen II, cit-histones, and cit-vimentin. The first two are not present in ECV304 cells. Pept-histones and cit-pept-histones were detected in both CTR and CIT-ECV304 lysates respectively, suggesting that native histones are also antigenic in RA as in other connective tissue diseases. Cit-vimentin, the Sa antigen was originally found in placenta and pannus lysates [19]. Vimentin is present in ECV304 [35] and in the CTR-ECV304 lysate. However, neither native nor cit-vimentin were present in the proteomic analysis. Vimentin was present in ECV304 cell lysates as detected by WB with the monoclonal anti-vimentin antibody that recognized vimentin both in its native and citrullinated forms in placenta lysates (Fig. S2). Cit-vimentin wasn’t detected in the ECV304 CIT cell lysates. Previously, for anti-Sa detection, ECV304 cell lysates were citrullinated for 3 h at 37˚C [36]. In this paper, we citrullinated for 18 h at 55˚C given those were the optimized conditions. However, these conditions likely result in cit-vimentin degradation (Fig. S2). The addition of three protease inhibitors did not prevent the loss of cit-vimentin in cit-ECV304 lysates but rescued cit-vimentin in cit-placenta. Under those experimental conditions, other proteins were citrullinated, not degraded and were still recognized by RA sera (Fig. S2B).

Other identified CIT-ECV304 ACPA targets are novel citrullinated targets of RA. Cathepsin D mRNA, for example, is differentially expressed in RA and OA synovial tissues [37]. Chloride intracellular channel protein 1 is overexpressed in cancer stem cells and implicated in carcinogenesis [38]. Nucleophosmin is immunogenic in systemic sclerosis [39] and in antiphospholipid syndrome [40]. Protein disulfide-isomerase A3 is recognized by heart-infiltrating-T-cell clones from rheumatic heart disease patients [41]. Finally, transketolase is immunogenic in multiple sclerosis [42].

The CIT-ECV304 ELISA is a multi-auto-cit-prot-ACPA ELISA and was used to identify relevant “private” epitope-carrying-proteins. It has a diagnostic specificity in RA comparable to anti-CCP2 and slightly superior to RF (95.2, 95 and 85%, respectively). Its sensitivity is lower than anti-CCP2 and RF (50, 70 and 70%, respectively). This could support the hypothesis that each RA patient’s serum may favour a limited set of “private” cit-protein epitopes, but reacts more with CCP, a “promiscuous” widely cross-reactive cit-peptide. It would also be consistent with RF, being a prevalent secondary autoantibody system in a disease where many currently undetected autoimmune systems exist.

Future research should aim at documenting the coexistence of the two types of ACPAs: the less pathogenic promiscuous cit-pept-ACPAs currently used in the clinic and the more pathogenic private cit-prot-ACPAs [8]. Even if both types of ACPAs are sensitive and specific for RA diagnosis, they may not provide the same basic and clinical information in individual patients. The amount of circulating cit-pept-ACPAs usually remains stable for years in most patients, while that of the cit-prot-ACPAs might increase during flares or decrease during periods of remission [43]. Using proteomics, ECV304 could allow defining the order of appearance of specific cit-prot-ACPAs in pre-RA, document changes during the transition to clinical RA, and inform on which, if any, cit-prot-ACPAs should be monitored during the disease course.

We validated that ECV304 has several autoantigens, including cit-antigens recognized by RA and that these can be identified post-citrullination of the lysate utilizing IP with RA sera and MS analysis. The cit-antigens were produced by PAD2 and PAD3; however, evaluating novel antigens produced by the other PAD isotypes would be of interest. Several cell lines express one or more of the PAD isotypes in abundance. Therefore, the same approach used here could be applied to other cell lines with high levels of PAD1, PAD2, PAD3, PAD4 or PAD6.

The different proteins identified by RA sera highlight the complexity of the cit-prot-ACPAs. Our work provides a platform to facilitate understanding why different cit-autoantigens and ACPAs occur in individual patients, and document the role of cit-proteins-ACPAs in the course of RA [10].

## Conclusions

ECV304 cells overexpress PAD2 and PAD3 capable to citrullinate auto-proteins that are recognized by RA sera, informing novel, and known private cit-protein targets. A cit-ECV304 ELISA could be used to screen for ACPAs to many private cit-protein epitopes without the need of having an ELISA for each cit-protein. In selected patients, during the pre-RA to clinical RA phase, using proteomics on RA immunoprecipitates of the autocitrullinome of ECV304 might allow the specific identification of potentially critical private cit-epitopes, thus providing new insights into the putative role of ACPAs in RA pathogenesis. The ECV304 cell line is a promising tool to study citrullination and its inhibition/modulation by anti-rheumatic drugs.

## Supplementary Information

**Additional file 1: Fig. S1.** Time course of PAD activity in ECV304 cells by WB. ECV304 lysates (2µg of proteins/lane) were incubated at 37˚C in the presence of calcium (10 mM). Citrullinated proteins were detected by anti-citrulline (modified) detection kit. The reaction was stopped by adding EDTA (100 mM). PAD activity from ECV304 lysate can generate cit-proteins *in vitro*. No cit-proteins were present at time point 0.

**Additional file 2: Fig. S2.** Detection of vimentin and citrullinated vimentin by WB. (A) WB using anti-vimentin monoclonal clone V-9 (Sigma-Aldrich, St. Louis, MO, USA), showing the presence of vimentin in placenta lysate and ECV304 cell lysate (5µg protein/well). (B) WB using RA serum, showing the presence of citrullinated proteins in ECV304 cell lysate containing proteases inhibitors. The ECV304 containing protease arrest, calpain inhibitor and acetyl-calpastatin (homologous to the natural specific inhibitor of calpains) showed the presence of citrullinated proteins. The inhibitors were added before the citrullination. Inhibitors: 1 – Protein Arrest reagent (1X) (Calbiochem); 2 – Calpain inhibitor VI (1 µM) (Sigma-Aldrich) and 3 – Acetyl-Calpastatin (1 µM) (Sigma-Aldrich).

**Additional file 3: Table S1.** Primers used for identification of PAD isoenzymes.

**Additional file 4: Table S2.** Authentication of the ECV304 (ATCC CRL-1998) and T24 (ATC HTB-4) cell lines by Short Tandem Repeated DNA profiling at ATCC.

**Additional file 5: Table S3.** Proteins unique to CIT-ECV304 immunoprecipitates.

## Data Availability

The datasets used and/or analysed during the current study are available from the corresponding author on reasonable request.

## Abbreviations

ACPA: Anti-citrullinated protein antibodies
ACR: American College of Rheumatology
amu: Atomic mass units
ANCA: Antineutrophil cytoplasmic antibodies
ATCC: American Type Culture Collection
BSA: Bovine serum albumin
CCP2: Anti-cyclic-citrullinated-peptide 2
Cit-: Citrullinated
CIT: Autocitrullinated
CRP: C-reactive protein
CTR: Non-citrullinated control
DMARD: Disease-modifying antirheumatic drug
DTT: Dithiothreitol
EDTA: Ethylene diamine tetraacetic acid
ELISA: Enzyme-linked immunosorbent assay
EULAR: European League of Associations for Rheumatology
HCD: Higher-energy collisional dissociation
HSP: Heat shock protein
IgG: Immunoglobulin G
ILD: Interstitial lung disease
IP: Immunoprecipitation
MS: Mass spectrometry
OD: Optical density
OR: Odds Ratio
PAD: Peptidylarginine deiminase
PBS: Phosphate-buffered saline
Pept-: Peptide
Prot-: Protein
PVDF: Polyvinylidene fluoride
RA: Rheumatoid arthritis
RAPID-3: Routine Assessment of Patient Index Data 3
RI-MUHC: Research Institute of the McGill University Health Centre
RF: Rheumatoid factor
ROC: Receiver operating characteristic
RT: Room temperature
RT-PCR: Reverse transcription polymerase chain reaction
WB: Western blot
