# Supplementary figures and images for: Characterization and use of the ECV304 autoantigenic citrullinome to understand anti-citrullinated protein/peptide autoantibodies in rheumatoid arthritis
Source: Arthritis Res Ther. 2022 Jan 13;24:23. doi: 10.1186/s13075-021-02698-2 (PMC8756661; doi:10.1186/s13075-021-02698-2)

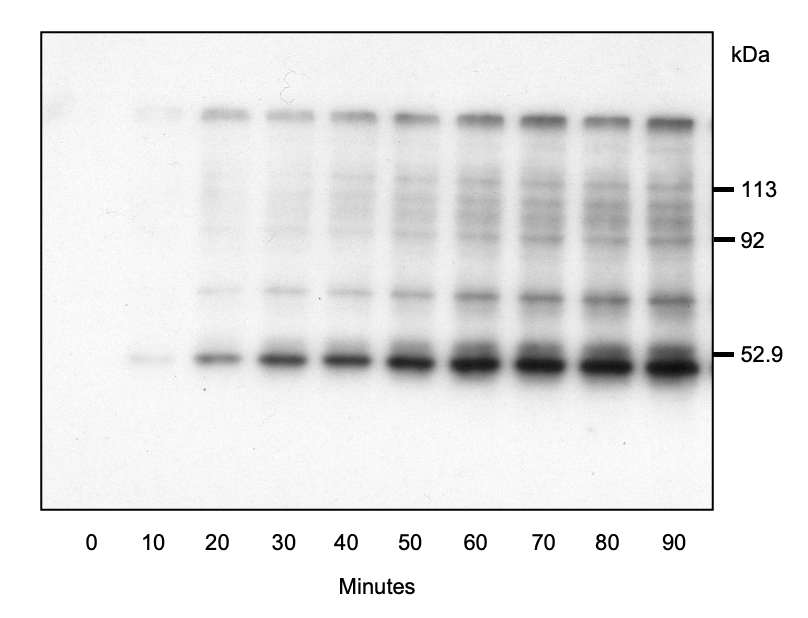

Supplement: Supplementary file 1 — Additional file 1: Fig. S1. Time course of PAD activity in ECV304 cells by WB. ECV304 lysates (2µg of proteins/lane) were incubated at 37˚C in the presence of calcium (10 mM). Citrullinated proteins were detected by anti-citrulline (modified) detection kit. The reaction was stopped by adding EDTA (100 mM). PAD activity from ECV304 lysate can generate cit-proteins in vitro. No cit-proteins were present at time point 0. [file 13075_2021_2698_MOESM1_ESM.tiff]

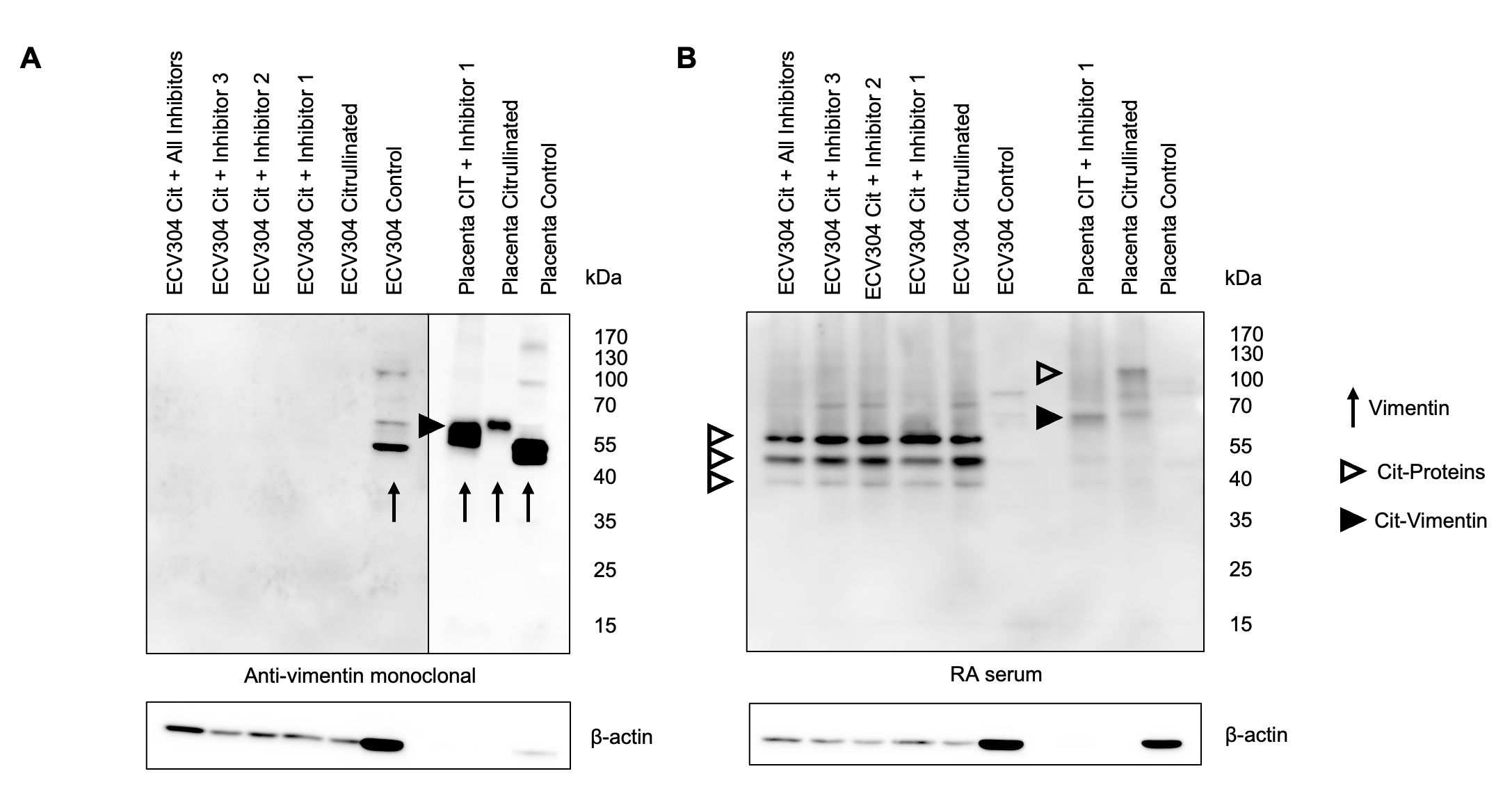

Supplement: Supplementary file 2 — Additional file 2: Fig. S2. Detection of vimentin and citrullinated vimentin by WB. (A) WB using anti-vimentin monoclonal clone V-9 (Sigma-Aldrich, St. Louis, MO, USA), showing the presence of vimentin in placenta lysate and ECV304 cell lysate (5µg protein/well). (B) WB using RA serum, showing the presence of citrullinated proteins in ECV304 cell lysate containing proteases inhibitors. The ECV304 containing protease arrest, calpain inhibitor and acetyl-calpastatin (homologous to the natural specific inhibitor of calpains) showed the presence of citrullinated proteins. The inhibitors were added before the citrullination. Inhibitors: 1 – Protein Arrest reagent (1X) (Calbiochem); 2 – Calpain inhibitor VI (1 µM) (Sigma-Aldrich) and 3 – Acetyl-Calpastatin (1 µM) (Sigma-Aldrich). [file 13075_2021_2698_MOESM2_ESM.tiff]
