# Supplementary material for: Characterization and use of the ECV304 autoantigenic citrullinome to understand anti-citrullinated protein/peptide autoantibodies in rheumatoid arthritis
Source: Arthritis Res Ther. 2022 Jan 13;24:23. doi: 10.1186/s13075-021-02698-2 (PMC8756661; doi:10.1186/s13075-021-02698-2)
Supplement: Supplementary file 3 — Additional file 3: Table S1. Primers used for identification of PAD isoenzymes. [file 13075_2021_2698_MOESM3_ESM.docx]

| *Table S1.* Primers used for identification of PAD isoenzymes | | | |  |
| --- | --- | --- | --- | --- |
| **Gene** | **Primer** | | **Amplicon size (bp)** | |
| PAD 1 | Forward | CGA GAT GGA GTT TGG CTA CA | 522 | |
|  | Reverse | GCT TCT TTT TGC CTG GTG TT |  |  |
| PAD 2 | Forward | GAC AAA GTG GGC GTG TTC TA | 586 | |
|  | Reserve | AGG CTG GTG ACA GAC TCA AA |  |  |
| PAD 3 | Forward | GAC CGA GAG GAA AAA AGC AA | 601 | |
|  | Reserve | AGA CCC CAA TGT GGA TTT GT |  |  |
| PAD 4 | Forward | AAG GAC TTC TTC ACA AAC CAT A | 596 | |
|  | Reserve | AAA ATC TGG ACC CAT CAC TC |  |  |
| GAPDH | Forward | CTT TGG TAT CGT GGA AGG AC | 488 | |
|  | Reserve | GTG TCG CTG TTG AAG TCA GA |  |  |
