# Supplementary material for: Characterization and use of the ECV304 autoantigenic citrullinome to understand anti-citrullinated protein/peptide autoantibodies in rheumatoid arthritis
Source: Arthritis Res Ther. 2022 Jan 13;24:23. doi: 10.1186/s13075-021-02698-2 (PMC8756661; doi:10.1186/s13075-021-02698-2)
Supplement: Supplementary file 4 — Additional file 4: Table S2. Authentication of the ECV304 (ATCC CRL-1998) and T24 (ATC HTB-4) cell lines by Short Tandem Repeated DNA profiling at ATCC. [file 13075_2021_2698_MOESM4_ESM.docx]

| *Table S2.* Authentication of the ECV304 (ATCC CRL-1998) and T24 (ATC HTB-4) cell lines by Short Tandem Repeated DNA profiling at ATCC | | | | | | | | | |  |
| --- | --- | --- | --- | --- | --- | --- | --- | --- | --- | --- |
| Designation | Short Tandem Repeats (STR) | | | | | | | | | |
|  | AMEL* | CSF1PO | D13S317 | D16S539 | D5S818 | D7S820 | THO1 | TPOX | vWA |  |
| T24^1^ | X | 10, 12 | 12 | 9 | 10, 12 | 10, 11 | 6 | 8, 11 | 17 |  |
| T24^2^ | X | 10, 12 | 12 | 9 | 10, 12 | 10, 11 | 6 | 8, 11 | 17 |  |
| ECV304^1^ | X | 12 | 12 | 9 | 10 | 10,11 | 6 | 8, 11 | 17 |  |
| ECV304^2^ | X | 12 | 12 | 9 | 10 | 10 | 6 | 8, 11 | 17 |  |

^*^AMEL: amelogenin

T24^1^: ATCC reference database profile (ATCC HTB-4); T24^2^: our cell line; ECV304^1^: ATCC reference database profile (ATCC CRL-1998); and ECV304^2^: our cell line.
