# Supplementary material for: Characterization and use of the ECV304 autoantigenic citrullinome to understand anti-citrullinated protein/peptide autoantibodies in rheumatoid arthritis
Source: Arthritis Res Ther. 2022 Jan 13;24:23. doi: 10.1186/s13075-021-02698-2 (PMC8756661; doi:10.1186/s13075-021-02698-2)
Supplement: Supplementary file 5 — Additional file 5: Table S3. Proteins unique to CIT-ECV304 immunoprecipitates. [file 13075_2021_2698_MOESM5_ESM.docx]

| *Table S3.* Proteins unique to CIT ECV304 immunoprecipitates | | |
| --- | --- | --- |
| Accession number | Name of Protein | Cit-peptides (coverage %) |
| ***1433B*** | ***14-3-3 protein beta/alpha*** | ***Yes (23)*** |
| ***1433E*** | ***14-3-3 protein epsilon*** | ***Yes (9.8)*** |
| ***1433Z*** | ***14-3-3 protein zeta/delta*** | ***Yes (18)*** |
| 6PGD | 6-phosphogluconate dehydrogenase, decarboxylating | No (4.6) |
| ACADV | Very long-chain specific acyl-CoA dehydrogenase, mitochondrial | Yes (5.3) |
| *ACTN1* | *Alpha-actinin-1* | *No (7.6)* |
| ***ALDOA*** | ***Fructose-bisphosphate aldolase A*** | ***Yes (20)*** |
| AMPL | Cytosol aminopeptidase | Yes (5.8) |
| ANXA1 | Annexin A1 | No (19) |
| ARPC3 | Actin-related protein 2/3 complex subunit 3 | Yes (16) |
| ***CALR*** | ***Calreticulin*** | ***Yes (3.4)*** |
| *CALX* | *Calnexin* | *No (7.6)* |
| CAND1 | Cullin-associated NEDD8-dissociated protein 1 | Yes (2.7) |
| *CAP1* | *Adenylyl cyclase-associated protein 1* | *Yes (13)* |
| ***CATD*** | ***Cathepsin D*** | ***Yes (10)*** |
| CBPN | Carboxypeptidase N catalytic chain | No (7.9) |
| CBX3 | Chromobox protein homolog 3 | No (15) |
| CDC42 | Cell division control protein 42 homolog | No (16) |
| CFAB | Complement factor B | No (9.6) |
| CH10 | 10 kDa heat shock protein, mitochondrial | No (25) |
| ***CLIC1*** | ***Chloride intracellular channel protein 1*** | ***No (12)*** |
| *COF1* | *Cofilin-1* | *Yes (56)* |
| *EF1D* | *Elongation factor 1-delta* | *No (19)* |
| ERP29 | Endoplasmic reticulum resident protein 29 | No (8.4) |
| ETFA | Electron transfer flavoprotein subunit alpha, mitochondrial | No (11) |
| *FLNA* | *Filamin-A* | *Yes (8.7)* |
| FLNB | Filamin-B | Yes (6.3) |
| FSCN1 | Fascin | No (3.9) |
| G6PI | Glucose-6-phosphate isomerase | Yes (17) |
| *GELS* | *Gelsolin* | *No (5.9)* |
| GNS | N-acetylglucosamine-6-sulfatase | No (4.7) |
| *GSTP1* | *Glutathione S-transferase P* | *No (41)* |
| HINT1 | Histidine triad nucleotide-binding protein 1 | Yes (32) |
| *HMGB1* | *High mobility group protein B1* | *No (16)* |
| HNRPL | Heterogeneous nuclear ribonucleoprotein L | Yes (14) |
| HSP74 | Heat shock 70 kDa protein 4 | No (5.7) |
| IF5A1 | Eukaryotic translation initiation factor 5A-1 | Yes (16) |
| *IMDH2* | *Inosine-5'-monophosphate dehydrogenase 2* | *Yes (7.6)* |
| *INO1* | *Inositol-3-phosphate synthase 1* | *Yes (6.8)* |
| IQGA1 | Ras GTPase-activating-like protein IQGAP1 | No (1.3) |
| KRT81 | Keratin, type II cuticular Hb1 | Yes (7.1) |
| KTN1 | Kinectin | Yes (7.2) |
| LA | Lupus La protein | No (6.6) |
| LMNB1 | Lamin-B1 | Yes (6.8) |
| MARE1 | Microtubule-associated protein RP/EB family member 1 | Yes (15) |
| *MDHC* | *Malate dehydrogenase, cytoplasmic* | No (23) |
| *MDHM* | *Malate dehydrogenase, mitochondrial* | *Yes (28)* |
| NASP | Nuclear autoantigenic sperm protein | No (3.0) |
| ***NPM*** | ***Nucleophosmin*** | ***No (23)*** |
| NTF2 | Nuclear transport factor 2 | No (11) |
| *OLA1* | *Obg-like ATPase 1* | *No (6.3)* |
| *PDIA1* | *Protein disulfide-isomerase* | *Yes (32)* |
| ***PDIA3*** | ***Protein disulfide-isomerase*** | ***Yes (25)*** |
| *PDIA4* | *Protein disulfide-isomerase* | *Yes (18)* |
| *PDIA6* | *Protein disulfide-isomerase A6* | *No (12)* |
| PGAM1 | Phosphoglycerate mutase 1 | Yes (52) |
| *PPIA* | *Peptidyl-prolyl cis-trans isomerase A* | *Yes (53)* |
| PPIB | Peptidyl-prolyl cis-trans isomerase B | No (28) |
| ***PRDX5*** | ***Peroxiredoxin-5, mitochondrial*** | ***Yes (19)*** |
| *PROF1* | *Profilin-1* | *No (56)* |
| PRP19 | Pre-mRNA-processing factor 19 | Yes (5.4) |
| PSA | Puromycin-sensitive aminopeptidase | No (4.5) |
| PSA2 | Proteasome subunit alpha type 2 | Yes (25) |
| PUR9 | Bifunctional purine biosynthesis protein PURH | No (4.4) |
| *RALY* | *RNA-binding protein Raly* | *Yes (6.5)* |
| RB11A | Ras-related protein Rab-11A | No (9.3) |
| *RL5* | *60S ribosomal protein L5* | *Yes (10)* |
| RL7A | 60S ribosomal protein L7a | Yes (7.9) |
| *RL8* | *60S ribosomal protein L8* | *Yes (7.8)* |
| RS19 | 40S ribosomal protein S19 | Yes (21) |
| RSSA | 40S ribosomal protein SA | No (7.1) |
| *SAHH* | *Adenosylhomocysteinases* | *No (15)* |
| SAP | Prosaposin | No (9.0) |
| SEPT2 | Septin-2 | No (8.6) |
| SEPT7 | Septin-7 | No (6.9) |
| SF3A3 | Splicing factor 3A subunit 3 | No (7.2) |
| SPTN1 | Spectrin alpha chain, non-erythrocytic 1 | No (1.9) |
| SYAC | Alanine--tRNA ligase, cytoplasmic | Yes (4.3) |
| SYEP | Bifunctional glutamate/proline--tRNA ligase | Yes (3.1) |
| SYHC | Histidine--tRNA ligase, cytoplasmic | No (2.9) |
| SYK | Lysine--tRNA ligase | No (4.0) |
| SYNC | Asparagine--tRNA ligase, cytoplasmic | No (2.6) |
| SYRC | Arginine--tRNA ligase, cytoplasmic | Yes (5.3) |
| SYSC | Serine--tRNA ligase, cytoplasmic | Yes (6.8) |
| *TALDO* | *Transaldolase* | *Yes (15)* |
| ***TERA*** | ***Transitional endoplasmic reticulum ATPase*** | ***Yes (33)*** |
| ***TKT*** | ***Transketolase*** | ***Yes (32)*** |
| TLN1 | Talin-1 | Yes (1.2) |
| *TMEDA* | *Transmembrane emp24 domain-containing protein 10* | *No (12)* |
| ***TPIS*** | ***Triosephosphate isomerase*** | ***No (30)*** |
| TXND5 | Thioredoxin domain-containing protein 5 | No (9.5) |
| UBA1 | Ubiquitin-like modifier-activating enzyme 1 | No (6.3) |
| *VDAC1* | *Voltage-dependent anion-selective channel protein 1* | *No (12)* |
| *VDAC2* | *Voltage-dependent anion-selective channel protein 2* | *No (10)* |
| *VINC* | *Vinculin* | *Yes (6.9)* |
| XRCC5 | X-ray repair cross-complementing protein 5 | No (4.5) |
| *XRCC6* | *X-ray repair cross-complementing protein 6* | *Yes (6.6)* |
